# Social functioning in individuals with Alzheimer's disease and the situation of caregivers

**DOI:** 10.1177/13872877251326029

**Published:** 2025-03-21

**Authors:** Sophia Kraake, Melanie Luppa, Dorothee Saur, Jens Dietzel, Jan-Philipp Bach, Steffi G Riedel-Heller, Janine Stein

**Affiliations:** 1 Institute of Social Medicine, Occupational Health and Public Health, Medical Faculty, University of Leipzig, Leipzig, Leipzig, Germany; 2 Department of Neurology, Neuroimaging Laboratory, University of Leipzig, Leipzig, Leipzig, Germany; 3 Department of Psychiatry and Psychotherapy, University of Leipzig Medical Centre, Leipzig, Leipzig, Germany; 4 Medical Practice for Neurology, Psychiatry, and Family Counseling, Gernsheim, Germany; 5 Department of Neurology, University Hospital of Gießen and Marburg (UKGM), Marburg site, Marburg, Germany

**Keywords:** Alzheimer's disease, caregiver burden, geriatrics, psychometrics

## Abstract

**Background:**

Changes in social functioning may be a significant parameter for the early detection of Alzheimer's disease (AD). Currently, research on social functioning in AD across the entire spectrum of the disease is lacking.

**Objective:**

The aim of this study was to describe the social functioning of persons with AD at each stage of the disease and to investigate how impaired social functioning affects caregiver burden.

**Methods:**

Cross-sectional data was derived from memory clinics across Germany as part of the pilot study “Social functioning in individuals with AD and the situation of caregivers”. A total of N = 87 relatives providing care for individuals with mild (n = 20), moderate (n = 40), and severe (n = 23) AD were included. Social functioning of individuals with AD was measured via the caregiver-rated German version of the Social Functioning in Dementia Scale (SF-DEM); caregiver burden was assessed using the Zarit Caregiver Burden Interview (ZBI-12). Differences between mild, moderate, and severe AD in terms of sociodemographic characteristics and the level of social functioning were examined. A robust linear regression analysis was conducted to examine the association between social functioning and caregiver burden.

**Results:**

Social functioning was lower in moderate and severe AD than in mild AD. Higher levels of social functioning were associated with less caregiver burden.

**Conclusions:**

This study highlights the importance of integrating social functioning assessments into clinical practice for improving the early detection, diagnosis and interventions for AD. Early interventions to enhance social functioning may diminish caregiver burden.

## Introduction

Alzheimer's disease (AD) is one of the most prevalent and severe illness in old age and has been demonstrated to considerably reduce life expectancy.^1^ In 2020, the global prevalence of individuals living with AD was estimated at over 55 million, with approximately 1.6 million cases in Germany.^2^ As the world's demographic changes result in a rapidly growing population of old people, with an increasing number of people with dementia, research is particularly important to gain a deeper understanding of the entire spectrum of AD. Apart from memory impairment, which represents the primary cognitive symptom of AD, other cognitive and behavioral symptoms emerge through the progression of the disease. Notably, changes in social functioning are often observed.^3^ In contrast to social behavior, social functioning is defined more broadly and encompasses a person's long-term and context-dependent abilities to interact with others.^4,5^ This includes, for example, how someone builds relationships with others, maintains them, and behaves within them. In this context, it is essential to consider the close relationship between the individual diagnosed with AD and their caregiver. Regarding AD, alterations in social functioning may manifest as a loss of interest in previously valued hobbies or withdrawal from social activities, which in turn may contribute to a deterioration in interpersonal relationships.^3,6^ The diagnosis of social functioning has been addressed with the inclusion of social cognition as a diagnostic criterion for dementia to the Diagnostic and Statistical Manual of Mental Disorders in 2018 (DSM-5). Prevalence rates indicate that up to two-thirds of individuals with AD receive care from relatives, a proportion that is still on the rise.^7,8^ A number of studies support the hypothesis that the impairment of social functioning in individuals with AD is associated with elevated stress levels among their caregiving relatives.^9–13^ This association may be attributed to several factors, including dysfunctional caregiver attitudes, social withdrawal of both the patient and the caregiver, challenging behavior and an increasing need for assistance among individuals with AD.^14–19^ The caregiver burden, in turn, may negatively influence the social functioning of the person with AD, thereby creating a self-reinforcing cycle.^20–22^ However, changes in social functioning are not routinely asked for or reported as a symptom in clinical settings. These circumstances highlight the necessity for the implementation of a validated assessment tool in both research and clinical practice.

A recently developed structured questionnaire, the Social Functioning in Dementia Scale (SF-DEM), has been designed to assess the level of social functioning in individuals with AD. This instrument was first introduced and validated in the Anglo-American region by Sommerlad et al.^6^ The original English version was subsequently translated into German in accordance with scientific standards and psychometrically validated as part of a pilot study by the authors.^23^ The SF-DEM consists of four sections that cover different aspects of social functioning and can be answered by both the person with AD and their caregiver. Social behavioral difficulties are evident in the earliest stages of the disease and can potentially disrupt interpersonal relations.^24^ In light of the aforementioned definitional similarity between social behavior and social functioning, one may hypothesize that social functioning may also serve as an indicator of the onset of dementia. In fact, a study conducted by Kotwal et al.^24^ revealed that social functioning may serve as an early indicator to screen for cognitive loss. Moreover, multiple longitudinal studies have shown that constructs that are likely to be associated with social functioning, such as the social network, social connections and access to social resources, may be a protective factor against a dementia diagnosis.^25–29^ It is therefore crucial to assess social functioning using the SF-DEM in order to improve early detection, diagnosis, the description of the course of the disease, and the evaluation of intervention effects.

To date, the German version of the SF-DEM has only been evaluated in a sample of individuals with mild dementia and their relatives.^23^ However, no research has been conducted in German-speaking countries to investigate the differences in social functioning across AD severity. Therefore, this exploratory study aims to provide cross-sectional data on social functioning in AD considering different stages of the disease in Germany. Another aim was to contrast the sociodemographic characteristics of the patient sample in mild, moderate, and severe stages of AD. In addition, the distribution of responses to the individual items on the SF-DEM will be described. Finally, the study aims at examining the relationship between the level of social functioning and caregiver burden.

## Methods

### Study design and sample

Data was collected via the pilot study “Social functioning in individuals with Alzheimer's disease (AD) and the situation of caregivers” as part of the program for the promotion of young scientists at the Faculty of Medicine at Leipzig University (formel.1). The aims of the project were to describe the level of social functioning as measured by the caregiver-rated version of the SF-DEM across each stage of the AD and to determine how impairments in social functioning affect caregiver burden. The objective of this study was to recruit a total sample of 90 caregivers of individuals with mild, moderate, and severe AD. For each level of severity, the targeted sample size was 30 caregivers of the patients.

The study participants were recruited from medical practices and from memory outpatient clinics throughout Germany that are specialized in the treatment of memory complaints. The patients were diagnosed with AD by their treating psychiatrist or geriatrician according to established criteria of the International Classification of Diseases and Related Health Problems, 10^th^ version (ICD-10 of WHO).^30^ Prior to the interview, all participants were informed about the aims and procedure of the study via an information sheet. Subsequently, participants were asked to provide their informed consent to take part in the study and to provide their contact details. Once the informed consent forms had been received, the staff from ISAP contacted the study participants by telephone to arrange appointments for the interviews. The structured interviews were conducted via telephone between January 2023 and April 2024 by trained interviewers. Criteria for inclusion into the study sample were that the caregivers were required to (1) be at least 18 years of age, (2) have at least one contact with the patients per week, (3) are unpaid, and to (4) possess at least an intermediate level of German language proficiency. Patients with serious physical or mental illness or insufficient capacity to consent were excluded from this study. If an SF-DEM assessment was incomplete or absent, the participant was also excluded from the sample.

**Figure 1.**
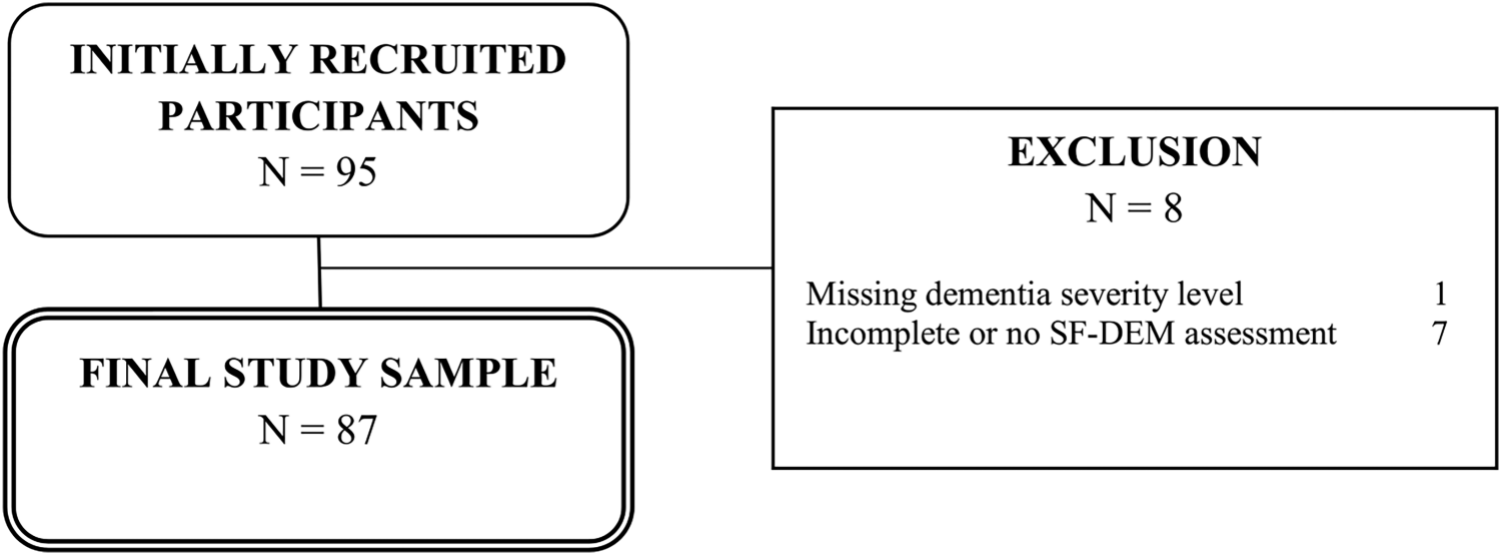
Flow chart of sample selection.

Figure 1 offers a comprehensive overview of the sample selection process. Initially, N = 95 caregivers were recruited and interviewed. Following the criteria for inclusion and exclusion, n = 8 participants had to be excluded from the sample, resulting in a final analytical sample size of 87 caregivers.

### Instruments

The structured telephone interviews included the German version of the Social Functioning in Dementia Scale (SF-DEM).^23^ This scale was translated from the original English version^6^ into German and psychometrically tested by the authors.^23^ It is a validated instrument for measuring social functioning in individuals with AD with an acceptable level of internal consistency as rated by the patients (α = 0.72) and by the caregivers (α = 0.76).^23^ In addition, Sommerlad et al.^6^ proved its content validity and concurrent validity against a single item rating overall social functioning. The SF-DEM comprises 20 items that are divided into four sections: “1. Spending time with other people”, “2. Communication with other people”, “3. Sensitivity to other people”, and “4. Global questions about SF-DEM”. The responses were scored on a 4-point Likert scale ranging from 0 (never) to 3 (very often). Sum scores were calculated based on the first three sections, comprising 17 items, with a range of 0 to 51. Higher scores indicate better social functioning. The items 12–17 were reverse coded and reverse-scored. The fourth section consisted of three questions. The first question was asking “thinking about their social life as a whole, how is it now?” and the responses scored on a 4-point Likert scale (excellent, good, fair, poor). The second question was asking “How is it now compared to 1 year ago?” and was rated on a 5-point Likert scale ranging from “a lot better” to “a lot worse”. Finally, the third question was asking “Would you like their social life to change?” and was rated on a 3-point Likert scale (rather do more, no change needed, rather do less).

Caregiver burden was assessed using the Zarit Burden Interview (ZBI-12), a well-established caregiver self-report measure. In this study, a short 12-item version of the ZBI with responses on a 5-point Likert scale ranging from 0 (never) to 4 (always) for each item was used.^31^ The total score ranges from 0 to 48, with higher scores indicating a greater caregiver burden. The ZBI-12 is a validated instrument designed to assess caregiver burden in community-dwelling older adults.^32^

A series of standardized questions were asked regarding the sociodemographic characteristics of both patients and their caregivers, including age (in years), gender (male/female), education, marital status (married, single/divorced, widowed), and domicile (alone in private household, living with partner/relatives/others, assisted living/living in institutions). The level of education was categorized as low/medium/high according to the new CASMIN classification of education.^33^

### Ethics

All study participants provided written informed consent prior to enrolment. This study was approved by the Ethics Committee of the Medical Faculty of the University of Leipzig (ref: 463/22-ek). The ethical standards of this study are consistent with the principles outlined in the Declaration of Helsinki of 1975 (as revised in 2008) and the relevant national and institutional committees on human experimentation.

### Statistical analyses

The statistical analysis was performed using Stata 16.0 SE (StataCorp LLC, College Station, TX). First, due to small subsample sizes, the marital status categories (married, single, divorced, widowed) “single” and “divorced” were collapsed into “single/divorced”. A similar approach was employed in the categorization of domicile types (living “alone in private household”, “together with partner”, “with relatives”, “with others in private household”, in “assisted livings”, “retirement home”, “nursing home”). This yielded three main categories: “alone in private household”, “living with partner/relatives/others” and “assisted living/living in institutions”. Based on this, the sociodemographic characteristics of the total sample of caregivers and patients were analyzed in terms of means, standard deviations, and percentages. In addition, differences in sociodemographic characteristics between mild, moderate, and severe AD patients were compared based on Fisher's exact test or Kruskal-Wallis test, as appropriate.

Descriptive statistics (percentages, mean, standard deviation, range) were calculated for the caregiver-rated scores on each item and the summary scores of sections 1, 2, and 3 of the SF-DEM. For the determination of the internal consistency of the SF-DEM, Cronbach's α was recorded for the total score and for each item in case it would be deleted. Furthermore, the group differences in mild, moderate, and severe AD of the SF-DEM total score as well as the summary scores of sections 1, 2, and 3 were contrasted using the Analysis of Variance (ANOVA).

A linear regression analysis was conducted to examine the relationship between the level of social functioning of the person with AD and caregiver burden. The analysis included AD severity as a covariate based on the established association with caregiver burden, while controlling for the sociodemographic characteristics age, gender, education, marital status and domicile of the patients. The Wald test was employed to assess the association of nominal predictor variables with more than two categories. The significance level was set at α ≤ 0.05 for all calculations.

## Results

### Characteristics of the caregiver and patient sample

The sociodemographic characteristics of both the caregiver and the patient samples are presented in Table 1. The mean age of the caregivers was lower (*M *= 66.6; *SD *= 11.1) compared to the mean age of patients (*M *= 79.3; *SD *= 7.3). Approximately two-thirds of the caregivers were female, while the gender of the patients was equally distributed. Overall, the caregivers exhibited a higher educational level than the patients. The majority of the surveyed caregivers (75.9%) and patients (70.1%) were married and most resided with a partner, relatives, or others (87.4% and 72.4%, respectively). Furthermore, the characteristics of the patient sample were described and compared based on the severity level of Alzheimer's disease (AD) (mild, moderate, severe). The majority of patients (50.6%) was diagnosed with moderate AD, while nearly one-quarter (23.0%) had mild AD and another quarter (26.4%) had severe AD. A significant correlation was identified between the level of education and the severity of AD (*p *= 0.015). Individuals with lower levels of education exhibited more severe AD compared to individuals with a higher educational level. The analysis revealed non-significant associations between the variables of age, gender, marital status, or domicile and the severity of AD.

**Table 1.** Sociodemographic characteristics of the caregivers and patient sample.

Variables	Caregivers	Patients				
	Total sample (N = 87)	Total sample (N = 87)	mild AD^a^ (*n *= 20, 23.0%)	moderate AD (*n *= 44, 50.6%)	severe AD (*n *= 23, 26.4%)	*p* ^b^
**Age**						
Mean (SD)	66.6 (11.1)	79.3 (7.3)	78.7 (6.0)	79.1 (7.8)	80.3 (7.6)	0.425
Range	38–89	54–93	65–89	54–92	60–93	
**Gender (n, (%))**						
Male	27 (31.0)	44 (50.6)	10 (50.0)	20 (45.5)	14 (60.9)	0.480
Female	60 (69.0)	43 (49.4)	10 (50.0)	24 (54.5)	9 (39.1)	
**Education^c^ (n, (%))**						
High	27 (31.0)	21 (24.1)	8 (40.0)	12 (27.3)	1 (4.4)	**0**.**015**
Middle	47 (54.0)	32 (36.8)	7 (35.0)	18 (40.9)	7 (30.4)	
Low	13 (15.0)	34 (39.1)	5 (25.0)	14 (31.8)	15 (65.2)	
**Marital Status (n, (%))**						
Married	66 (75.9)	61 (70.1)	14 (70.0)	30 (68.2)	17 (73.9)	0.729
Single/Divorced	18 (20.7)	5 (5.8)	2 (10.0)	3 (6.8)	0 (0.0)	
Widowed	3 (3.5)	21 (24.1)	4 (20.0)	11 (25.0)	6 (26.1)	
**Domicile (n, (%))**						
Alone in private household	11 (12.6)	17 (19.5)	5 (25.0)	10 (22.7)	2 (8.7)	0.073
Living together with partner/relatives/others	76 (87.4)	63 (72.4)	15 (75.0)	32 (72.7)	16 (69.6)	
Assisted living/Living in institutions	0 (0.0)	7 (8.1)	0 (0.0)	2 (4.6)	5 (21.7)	

N/*n*: sample size; SD: standard deviation; ^a^AD: Alzheimer's disease; ^b^comparison of mild, moderate, and severe AD patients is based on Fisher's exact test or Kruskal-Wallis-test, as appropriate; ^c^educational classification according to the new CASMIN educational classification. Low: inadequately completed general education, general elementary education, basic vocational qualification or general elementary education and vocational qualification; Middle: intermediate vocational qualification or intermediate general qualification and vocational qualification, intermediate general qualification, general maturity certificate, vocational maturity certificate/general maturity certificate and vocational qualification; High: lower tertiary education - general diplomas/diplomas with vocational emphasis, higher tertiary education – lower level/higher level. Bold values represent statistically significant results at α < 0.05.

### SF-DEM

Table 2 presents the frequency and percentage distributions of caregiver-rated responses to each SF-DEM item. The full range of possible responses was utilized by the caregivers of people with AD for 15 out of 17 questions of the SF-DEM. A majority of respondents indicated similar responses to a number of items on the survey. Approximately half of the caregivers indicated that the patients have often “seen friends or family in own home” (52.9%, item 1) and have often “gone shopping with friends or family” (49.4%, item 4). More than half stated they have never “attended community or religious meetings” (86.2%, item 3) and never “gone on trips or events like the cinema or talks” (62.1%, item 5). Moreover, most caregivers rated the person with AD has very often “started or taken part in a conversation” (60.9%, item 9). The majority indicated that people with AD never “asked other people about their feelings or concerns” (56.3%, item 11), never “found it difficult to think of something to say to others” (57.5%, item 12), never “found that other people are irritating” (51.7%, item 15), and never “had an argument or shouted at other people” (67.8%, item 16). The summary scores of each section revealed that section 2 “Communication with other people” had the highest mean value (*M *= 8.6, *SD *= 3.9), compared to sections 1 and 3. In section 4, entitled “Global questions about SF-DEM”, 48% of the caregivers indicated that their relatives’ social life as a whole was “fair”. 42.4% indicated that their relatives’ social life was “a bit worse” compared to 1 year ago, and 67.8% liked their relatives to engage in social life to a greater extent (“rather do more”).

**Table 2.** Caregiver-rated scores on SF-DEM.

	Caregiver-rated (N = 87)				
**Items**	**Frequency (*n* (%))**				
SF-DEM domain: How often in the past month have they…	Very often (0)	Often (1)	Occasionally (2)	Never (3)	Cronbach's α if item deleted
**Spending time with other people**					
1. Seen friends or family in own home	25 (28.7)	46 (52.9)	9 (10.3)	7 (8.1)	0.65
2. Visited friends or family at their homes	3 (3.5)	24 (27.5)	30 (34.5)	30 (34.5)	0.64
3. Attended community or religious meetings	0 (0)	5 (5.8)	7 (8.1)	75 (86.1)	0.67
4. Gone shopping with friends or family	1 (1.2)	43 (49.4)	17 (19.5)	26 (29.9)	0.63
5. Gone on trips or events like the cinema or talks	0 (0)	15 (17.2)	18 (20.7)	54 (62.1)	0.62
6. Gone to a café, restaurant, pub, or social club	3 (3.5)	31 (35.6)	27 (31.0)	26 (29.9)	0.63
7. Exercised, walked, or played sport with others	15 (17.2)	35 (40.3)	9 (10.3)	28 (32.2)	0.64
**Communication with other people**					
8. Contacted friends or family by phone or computer	8 (9.2)	33 (37.9)	13 (14.9)	33 (37.9)	0.61
9. Started or taken part in a conversation	53 (60.9)	19 (21.8)	3 (3.5)	12 (13.8)	0.63
10. Talked to others about your/their feelings or concerns	11 (12.6)	26 (29.9)	11 (12.6)	39 (44.8)	0.64
11. Asked other people about their feelings or concerns	3 (3.5)	23 (26.4)	12 (13.8)	49 (56.3)	0.62
12. Found it difficult to think of something to say to others	14 (16.1)	14 (16.1)	9 (10.3)	50 (57.5)	0.65
13. Found other people's conversation unclear	23 (26.4)	35 (40.2)	18 (20.7)	11 (12.6)	0.64
**Sensitivity to other people**					
14. Been outspoken about what you/they really think	5 (5.8)	30 (34.5)	15 (17.2)	37 (42.5)	0.69
15. Found that other people are irritating	3 (3.5)	25 (28.7)	14 (16.1)	45 (51.7)	0.66
16. Had an argument or shouted at other people	1 (1.2)	17 (19.5)	10 (11.5)	59 (67.8)	0.68
17. Found reasons not to do things you/they would usually do	17 (19.5)	27 (31.0)	10 (11.5)	33 (37.9)	0.68

**Cronbach's α for total score**				**0.66**	
	Mean (SD)		Range		
**Summary Scores**					
Section 1 “Spending time with other people” (1–7)	7.5 (3.4)		2–17		
Section 2 “Communicating with other people” (8–13)	8.6 (3.9)		0–15		
Section 3 “Sensibility to other people” (14–17)	8.3 (2.6)		1–12		
Total	24.4 (6.6)		7–37		

	Caregiver-rated (N = 87)				
	**Frequency (*n* (%))**				
	Excellent (1)	Good (2)	Fair (3)	Poor (4)	
Thinking about their social life as a whole, how is it now?	2 (2.3)	20 (23.0)	42 (48.3)	23 (26.4)	
	A lot better (1)	A bit better (2)	No change (3)	A bit worse (4)	A lot worse (5)
How is it now compared to 1 year ago?	2 (2.3)	2 (2.3)	18 (20.7)	37 (42.5)	28 (32.2)
	Rather do more	No change needed		Rather do less	
Would you like their social life to change?*	59 (67.8)	24 (27.6)		3 (3.5)	

N/*n*: sample size; SD: standard deviation; SF-DEM: Social functioning in Dementia Scale; * missing values: n = 1; For each question, higher score indicates better social functioning. For questions 12–17: reverse scored.

Cronbach's α as a term for the internal consistency for the total score of the SF-DEM was at an acceptable level (α = 0.66).^34^ The Cronbach's α coefficient increased when items 3, 14, 16, and 17 were removed, and it decreased when the remaining items were deleted.

As shown in Table 3, the overall rating of social functioning across the 17 items by caregivers differed significantly between individuals with mild, moderate, and severe AD (*F_total_*(2,84) = 21.4, *p *< 0.01). People with less severe AD were rated with higher scores on the SF-DEM, indicating a higher level of social functioning. A comparable pattern was observed for sections 1, 2, and 3 of the SF-DEM, with higher scores indicating a milder level of AD. A comparison of the mean scores for each section revealed statistically significant differences in the severity levels of AD (*F_section1_*(2,84) = 4.0, *p *= 0.02; *F_section2_*(2,84) = 23.4, *p *< 0.01; *F_section3_*(2,84) = 4.2, *p *= 0.02). A closer look into each SF-DEM item indicated that the restrictions in social functioning in moderate AD were evident in item 3 (“Attended community or religious meetings” with 88.64% who were rated as “never”), item 5 (“Gone on trips or events like the cinema or talks” with 56.82% who were rated as “never”), item 11 (“Asked other people about their feelings or concerns” with 52.27% who were rated as “never”), and item 13 (“Found other people's conversation unclear” with 40.91% who were rated as “often”). The restrictions in social functioning in severe AD were evident in item 3 (“Attended community or religious meetings” with 86.96% who were rated as “never”), item 4 (“Gone shopping with friends or family” with 56.52% who were rated as “never”), item 5 (“Gone on trips or events like the cinema or talks” with 82.61% who were rated as “never”), item 8 (“Contacted friends or family by phone or computer” with 82.61% who were rated as “never), item 10 (“Talked to others about your/their feelings or concerns” with 65.22% who were rated as “never”), item 11 (“Asked other people about their feelings or concerns” with 82.61% who were rated as “never”), item 13 (“Found other people's conversation unclear” with 47.83% who were rated as “very often”), item 14 (“Been outspoken about what you/they really think” with 47.83% who were rated as “never”), and item 15 (“Found that other people are irritating” with 43.48% who were rated as “often”).

**Table 3.** Differences in social functioning across Alzheimer's dementia severity levels.

**SF-DEM^a^** (Caregiver-rated)	**Patients** (N = 87)				
	**mild AD^b^** (*n *= 20, 23.0%)	**moderate AD** (*n *= 44, 50.6%)	**severe AD** (*n *= 23, 26.4%)	**Test statistics^c^**	***p***
**Total score (**Mean (SD))	29.9 (5.1)	24.8 (6.4)	19 (3.4)	F = 21.4	**<0**.**01**
**Spending time with other people (**Mean (SD))	9.2 (3.6)	7.4 (3.2)	6.3 (3.0)	F = 4.0	** 0**.**022**
**Communication with other people (**Mean (SD))	11.1 (2.9)	9.5 (3.4)	4.9 (3.0)	F = 23.4	** < 0**.**01**
**Sensitivity to other people (**Mean (SD))	9.7 (1.8)	7.9 (2.8)	7.8 (2.5)	F = 4.2	** 0**.**018**

N/*n*: sample size; SD: standard deviation; ^a^SF-DEM: Social functioning in Dementia Scale; ^b^AD: Alzheimer's disease; ^c^ANOVA; Bold values represent statistically significant results at α < 0.05.

### SF-DEM and caregiver burden

Table 4 presents the results of the linear regression analysis. A statistically significant association was observed between an increase in the SF-DEM total score and a decrease in the ZBI-12 total score reflecting caregiver burden (*B *= -0.495, *robust SE *= 0.16, *p *= 0.003). Non-significant associations between sociodemographic characteristics of the patients (age, gender, education, marital status) and the caregiver burden were observed, except for domicile. Here, we observed an overall significant association between domicile and the caregiver burden (Wald test *F* (2,75)*=* 4.4; *p *= 0.016). Specifically, living with partner/relatives/others in reference to living alone in private household was significantly associated with the caregiver burden measured via the ZBI-12 (*B *= -6.931, *robust SE *= 2.433, *p *= 0.006).

**Table 4.** Results of the linear regression analysis for cross-sectional prediction of caregiver burden.

Model-Variables^b^	ZBI-12^a^ (N = 87)				
	B	95% CI	robust SE	Wald/F	*p*
**SF-DEM total score^c^**	−0.495	−0.814 ; -0.176	0.160		**0**.**003**
**AD severity^d^**				2.14	0.124
* Moderate*	2.121	−2.386 ; 6.628	2.262		0.351
* Severe*	5.702	−0.036 ; 11.440	2.880		0.051
**Age**	−0.075	−0.301 ; 0.151	0.113		0.510
**Male**	−2.057	−6.099 ; 1.984	2.057		0.314
**Education^e^**				0.46	0.634
* Middle*	1.245	−3.045 ; 5.535	2.154		0.565
* High*	2.655	−2.873 ; 8.183	2.775		0.342
**Marital status^f^**				0.19	0.825
* Single/Divorced*	−0.379	−7.704 ; 6.946	3.677		0.918
* Widowed*	−1.736	−7.472 ; 3.999	2.879		0.548
**Domicile^g^**				4.40	**0**.**016**
* Living with partner/relatives/others*	−6.931	−11.777 ; -2.084	2.433		**0**.**006**
* Assisted living/ Living in institutions*	−6.417	−14.508 ; 1.673	4.061		0.118
					
* *Constant	37.064	15.775 ; 58.354	10.687		**0**.**001**

N: sample size; B: regression coefficient; F: F statistic; CI: confidence interval; robust SE: robust standard error; ^a^ZBI-12: Zarit Burden Interview; ^b^sociodemographic characteristics of the patient sample; ^c^SF-DEM: Social functioning in Dementia Scale; ^d^AD: Alzheimer's disease; Reference group for AD severity: mild; ^e^educational classification according to the new CASMIN educational classification. Low: inadequately completed general education, general elementary education, basic vocational qualification or general elementary education and vocational qualification; Middle: intermediate vocational qualification or intermediate general qualification and vocational qualification, intermediate general qualification, general maturity certificate, vocational maturity certificate/general maturity certificate and vocational qualification; High: lower tertiary education - general diplomas/diplomas with vocational emphasis, higher tertiary education – lower level/higher level. Reference group for education: low. ^f^Reference group for marital status: married; ^g^Reference group for domicile: alone in private household. Bold values represent statistically significant results at α < 0.05.

## Discussion

In the current study, our primary objective was to analyze the association between the level of social functioning across all stages of AD and the caregiver burden in a nationwide German sample of caregivers of older adults with AD. As a main result, we found that higher levels of social functioning in individuals with AD were related to less caregiver burden among their relatives. Besides social functioning, caregivers of patients residing with their partners, relatives, or others reported less caregiver burden compared to caregivers of patients living alone.

### The SF-DEM scale and its characteristics across AD severity

A psychometric evaluation of the SF-DEM scale demonstrated that the scale adequately reflects social functioning in patients with AD as an underlying cohesive construct, as estimated by the coefficient α that represented an acceptable level of internal consistency.^34^ The mean values of the SF-DEM scale as rated by the caregivers in this study sample were found to be lower than the caregiver-rated mean value observed in previous studies on social functioning in dementia by Sommerlad et al.^6^ and Grothe et al..^23^ In comparison with the study conducted by Grothe et al.,^23^ the average scores were lower in our study for sections 1, 2, and 3 of the SF-DEM, with the largest discrepancy observed in section 2, “Communication to other people”. A direct comparison of our SF-DEM scale to the scores across each SF-DEM section in the study conducted by Sommerlad et al.^6^ was not feasible, since the SF-DEM scale was divided into only two sections. Overall, the differences in the SF-DEM mean values may be explained by the fact that both the studies by Grothe et al.^23^ and Sommerlad et al.^6^ exclusively focused on patients with mild dementia, whereas the present study included AD across mild, moderate, and severe levels of severity. Our findings indicated that social functioning varied considerably across the spectrum of AD severity. In particular, individuals with more severe forms of AD tended to have lower social functioning. This finding is consistent with the results of the multicenter study conducted by Budgett et al.,^35^ which examined the psychometric properties of the SF-DEM across mild, moderate, and severe dementia, as well as the SF-DEM scores between the levels of dementia severity. A more comprehensive analysis of each SF-DEM item in moderate and severe AD revealed that in individuals with moderate AD, the restrictions in social functioning are particularly evident in the participation in daily activities and community meetings, as well as in communication with others (item 3, 5, 11, and 13). In individuals with severe AD, there were additional and more incisive restrictions in social functioning, including an inability to utilize a telephone or computer to contact friends or family, as well as a more restricted capacity for communication and understanding others (item 3, 4, 5, 8, 10, 11, 13, 14, 15). As AD progresses, people may become less engaged in social activities. As described above, this may be attributed to a decline in communicative abilities, which may lead to feelings of irritation, shame or fear of stigmatization among those affected and which may be distressing for friends and family.^36,37^ This, in turn, may result in a reduction of social visits, thereby increasing the social isolation of people with AD. In addition, social norms restrict the acceptance of increasingly challenging behaviors and agitation that accompany the progression of the disease, potentially leading to a growing distance between the individual and their social network. 38

The sociodemographic analysis of AD severity revealed significant variation in the educational level among patients across the AD severity levels. Higher education was found to be associated with milder AD, a finding that is consistent with the results of prior research studies in this field.^36,39–41^ Theories attempting to provide an explanation for this relationship hypothesize that higher education fosters the development of cognitive reserves, suggesting that the brain has a higher cognitive adaptability to counteract the neuropathology linked with dementia.^40,42,43^ This increased cognitive adaptability may also influence various aspects of patient care and outcomes, including the social functioning of patients diagnosed with AD. However, further research is required to investigate this hypothesis.

### Impaired social functioning and the caregiver burden

The aforementioned decline in social functioning among individuals with progressing AD has a significant impact on the caregiver burden, as evidenced by our findings. Lower social functioning was associated with a higher caregiver burden and this finding is consistent with prior research in this field.^9–12^ The rationale behind this association is likely to be complex and may be based on several factors. As social functioning declines, patients may become increasingly dependent on their immediate environment, particularly on their caregivers. This increased dependence may be observed in the context of daily activities, social interaction, communicative skills, and behavioral difficulties.^14,38,44^ Our evaluation of the SF-DEM scale demonstrated that in each section, namely “Spending time with other people”, “Communication with other people”, and “Sensitivity to other people”, there is a significant decline in social functioning as the severity of the disease advances. The deterioration of the communicative abilities may present a barrier for the caregiver to understand and address the needs of the individual with AD.^45^ Challenging behaviors such as agitation, aggression, or disinhibition that may emerge in moderate and severe forms of AD can be difficult to handle.^14,46,47^ The anticipated loss of the individual's personal identity of the person with AD can be a highly frightening and emotionally draining experience for the caregiver.^48,49^ Consequently, caregivers may experience elevated levels of stress, fatigue and an increased caregiver burden. Another contributing factor to the caregiver burden may be attributed to a lack of knowledge regarding dementia, the progression of the disease and the skills for the provision of daily care.^50,51^ Psychoeducational approaches and multicomponent interventions, including information provision, support groups, communication skills training, and mindfulness-based techniques play a crucial role in the enhancement of health literacy among caregivers of patients with AD.^52–54^ In addition, a recent systematic review by Hofbauer et al.^55^ examined the efficacy of music-based interventions in 327 community-dwelling individuals with dementia. The findings suggest that music-based interventions may have an immediate positive influence on cognition. Furthermore, music-based interventions lasting 1 was 4 months were associated with less anxiety and pain and enhanced cognition.^55^ Nevertheless, accessing help and services may be difficult given the pervasive public stigma surrounding dementia.^56,57^ The stigma does not only affect those living with the condition but also extends to their caregivers.^58,59^ The public representation of AD is largely characterized by an emphasis on the devastating negative consequences and complex implications of the disease, which may foster a perception of fear and dysfunctional thoughts and attitudes about AD.^18,57,60,61^ This, in turn, may have a significant and enduring impact on the coping style of the caregiver, as well as on the social isolation of both the caregiver and the person with AD.^62^ As a result, the caregiver burden may be further exacerbated. Therefore, it is essential to disseminate comprehensive and destigmatizing information including educational and supportive interventions for both the caregivers and individuals affected by AD in the early stages of the disease.

Given the complex interrelationship between the caregiver and the patient with AD, a dyadic perspective may be helpful to enhance the understanding of the caregiver burden. The concept of social health offers a dynamic approach that aims to capture this dyadic perspective on health.^63^ It is defined as a dynamic balance between an individual's capabilities and the restrictions of their social environment. In this sense, social health is not characterized by a steady state but a reciprocal relational concept. The concept of social health may provide a theoretical basis for our finding that impaired social functioning in advanced stages of AD was associated with an increase in caregiver burden. As previously outlined, individuals with AD may exhibit profound deficiencies in their social functioning capabilities, particularly in the domains of social participation, communication skills and sensitivity to others.^6,23,35^ These impairments may interact with the restrictions of their social environment. Social health addresses the structural and functional aspects of the social environment, as well as the individual's appraisal of the social environment.^63^ The structural aspect includes the number, frequency, and nature of social contacts, as well as access to resources, which can vary considerably between individuals. A smaller network may increase caregiver burden, as caregivers may feel solely responsible for the care of the person with AD. The functional aspects include emotional and informational support. A lack of information about the disease may lead caregivers to become distressed by the altered behavior, to misinterpret their behavior, and to hold irrational expectations of themselves and the person with AD. The appraisal of the social environment, influenced by feelings of loneliness and stigmatization, may further increase the caregiver burden. 63

There is conflicting evidence on the effects of social interventions on cognition and little evidence on delaying or preventing AD.^64–67^ A innovative international development in community-based health care to promote social participation is offered by the evidence-based concept of social prescribing.^68^ Depending on the (unmet) social needs of the patient, the GP can refer the patient to a so-called link worker who matches the individual needs to appropriate social activities in their community.^69,70^ As such, social prescribing is an individualized and holistic approach that focuses on unmet social needs and may be helpful for the prevention of Alzheimer's disease. However, future research is needed for the development of potentially more intensive social interventions aimed at reducing the risk of AD in old age. 36,67

### The influence of cohabitation with AD patients on caregiver burden

Another finding was that the type of domicile of AD patients, particularly those living with a partner, relatives, or others, in comparison to AD patients living alone, was associated with a reduction in caregiver burden. However, this is inconsistent with the findings of previous studies.^44,71^ The inconsistency may be attributed to the fact that our study was limited to a small sample size, especially in the domains of “assisted living/living in institutions” and “living alone in private household”. This may have diminished the statistical power to detect a true effect, which may have subsequently biased the interpretation of our results. In comparison, the studies conducted by Park et al.^44^ and Brini et al.^71^ included a considerably larger sample size of AD patients with a total of N = 1133 and N = 240 dementia patients, respectively. Nevertheless, an explanation for our result may be derived from the dyadic social health perspective. On the one hand, living with an individual diagnosed with AD may enhance the caregiver's sense of control and may reduce feelings of anxiety and worry. On the other hand, an individual with AD may perceive a higher level of emotional support, which may reduce feelings of social isolation and loneliness. This positive dynamic may strengthen the relationship between the AD patient and the caregiver, thereby potentially reducing the caregiver burden. However, further research is necessary to ascertain the nature of the relationship between the type of domicile of AD patients and the caregiver burden.

### The role of AD severity in caregiver burden

Interestingly, AD severity was not significantly associated with caregiver burden. However, a marginally significant association was found in our analyses between severe AD, in contrast to mild AD, and the degree of caregiver burden. Considering the predictor variables included in our statistical model, the significant negative correlation between the level of social functioning and the caregiver burden may have already accounted for the caregiver burden that may be associated with severe AD. The close link between social functioning and AD severity is supported by our findings discussed above. Indeed, a distinct analysis (not shown) revealed a significant positive correlation between the severity of AD and the degree of caregiver burden when AD severity was identified as the sole predictor variable. Future studies may investigate the underlying mechanisms and a potential interaction between AD severity and social functioning, as well as its association with caregiver burden.

### Strengths and limitations

This study succeeded in recruiting a relatively large sample of caregivers of AD patients aged 75 years and older across all stages of the disease. Given the difficulty of reaching this demographic sample, the size of this sample represents a notable strength of this study. The study participants were recruited from medical practices and memory outpatient clinics throughout Germany. Nevertheless, it is necessary to include a larger sample size in future studies in order to generalize the findings regarding the caregiver burden and social functioning of AD patients at all stages of the disease. It may be advisable to include caregiving relatives in all stages of the recruitment process, as recommended in the community-based participatory research (CBPR).^72^ Including caregiving relatives may help to increase the sample size of future studies while also providing the opportunity to investigate their care needs. Further, the interpretation of the level of social functioning in AD may be constrained by the fact that the SF-DEM does not fully control for the patient's social functioning prior to the AD diagnosis. However, the SF-DEM includes one item asking about the social life of the patient compared to one year ago. A longitudinal study design may facilitate a more profound understanding of the trajectory of social functioning over time.

### Conclusions and future directions

The study results highlighted the detrimental effect of impaired social functioning in the progression of AD on the caregiver burden. To facilitate the early detection, diagnosis, and intervention for AD, social functioning assessments such as the SF-DEM scale should be incorporated into clinical practice. The dynamic concept of social health may provide a useful framework for understanding and improving the relationship between impaired social functioning in the AD patients and the caregiver burden, with interventions targeting both the patients and the caregivers. Future research including a larger sample size is recommended to cross-validate our findings. In addition, longitudinal study designs may be helpful in accounting for prior social functioning and may provide a more accurate understanding of the relationship between social functioning and caregiver burden over time. Potential underlying mechanisms, such as economical stress, caregiver sex, and the coping strategy (emotional- versus problem-focused) may be further investigated in this regard.
